# Individuals Adjust Their Degree of Advice Taking to Impress Others

**DOI:** 10.1002/pchj.70082

**Published:** 2026-01-21

**Authors:** Xiufang Du, Shun Wu, Rui Li, Fen Ren

**Affiliations:** ^1^ School of Education and Psychology University of Jinan Jinan China; ^2^ School of Psychology Shandong Normal University Jinan China; ^3^ Inner Mongolia Student Bullying Prevention Research Center Tongliao China

**Keywords:** advice taking, competence, impression management, meta‐perception, warmth

## Abstract

As a form of interpersonal interaction, advice taking may occur after decision makers consider others' evaluations and impressions of them. Previous work has examined how other people view decision makers when they do or do not decide to take their advice. Our work examines whether decision makers realize this effect and therefore take advice strategically for the purpose of impression management. Four studies were designed to investigate the impression management mechanism of advice taking. Study 1 shows that individuals tend to consider decision makers who accepted others' advice as warmer and those who rejected others' advice as more competent. Decision makers can also accurately predict others' view of them in terms of whether they are warm or competent from their degree of advice taking. Studies 2 and 3 show that decision makers strategically adopt others' advice to engage in impression management. That is, when they want to show others they are warm, they rely more heavily on advice, whereas when they want to show they are competent, they rely less on advice. Study 4 shows that impression management in advice taking is more likely to occur when the advice comes from a person whom they want to impress.

## 1 Introduction

Whether in daily life or in terms of organizational management, people are faced with various decisions. Most of the time, decisions are not made by the decision maker alone. Rather, the decision maker often confers with one or more advisors to make decisions; this decision‐making process is called advice taking (Harvey and Fisher 1997; Yaniv and Kleinberger 2000). Generally, taking others' advice not only improves the chance of making a good decision (Harvey and Fisher 1997), but also has a positive effect on judges' subsequent initial judgments (Schultze et al. 2025). However, decision makers not only focus on making accurate decisions but also may consider what kinds of social assessments and impressions the decision‐making process will generate and may then take advice strategically as an impression management tool. Previous work in this respect has focused almost exclusively on advice taking and impression formation (Lee 2002; See et al. 2011; Tost et al. 2012). In contrast, we position our studies on the other side of this equation. The current work employs impression management theory as a theoretical framework to explore how advice‐taking serves as a strategic self‐presentation tool. Namely, while previous work has looked at how other people view decision makers when they do or do not take their advice, our work looks at whether decision makers recognize this effect and therefore take advice strategically as a means of impression management.

Our work makes several theoretical contributions. Firstly, the current research enriches the understanding of advice taking behaviours. Previous research on advice taking has focused on factors that influence advice taking behaviour (Gino et al. 2012; Sniezek and Van Swol 2001). However, little is known about the underlying reasons why decision makers adopt advice strategically. In this paper, we offer a new theoretical explanation for advice taking, that is, decision makers strategically adjust their degree of advice taking to engage in impression management. Secondly, in contrast to most extant advice research on impression formation, our study examines whether decision makers recognize the effect of advice taking and therefore take advice strategically as a means of impression management. In particular, our work focuses on the other side of this equation and provides deep insight into interpersonal impressions in advice taking.

### 1.1 Interpersonal Perspective on Advice‐Taking

Previous studies in advice taking have mainly focused on how decision‐makers can use advice to improve decision accuracy (Rader et al. 2017). While advice taking is essentially an interpersonal behaviour, advice takers care about more than simply making a correct decision. While making accurate decisions is important, other outcomes, such as future relationship between the advisor and advice taker and the impressions they make on the advisors, matter as well (Brooks et al. 2015; Goldsmith and Fitch 1997; Liljenquist 2010; Rader et al. 2017).

In recent years, researchers have begun to explore the influence of advice seeking, taking, or giving within interpersonal interactions and the self‐concept maintenance framework and have made many interesting findings (Palmeira and Spassova 2024; Rader et al. 2017). From the advisor's perspective, they occasionally interpersonally penalize seekers who disregard their advice and that these reactions are especially strong among expert advisors (Blunden et al. 2019). In repeated advice exchanges, a decision maker's rejection (vs. acceptance) of previous advice could reduce the advisor's prosocial motivation towards the decision maker and lead to dishonest (vs. honest) advice giving in a subsequent advising exchange (Belkin and Kong 2018). However, some studies found that advisors want their advice to be used but not too much. Instead, they often give advice about which they are uncertain and therefore want their advice to be averaged with the judge's initial opinions or not used at all, as a higher weighting might increase the advisor's perceived responsibility and accountability for the final judgment (Ache et al. 2020). From the decision maker's perspective, the purpose of advice taking may be to build a relationship or to derive benefits from the relationship. People may take advice to avoid damaging the relationship by not doing so (Goldsmith and Fitch 1997). Taking advice can serve relationship motives. Taking advice can also threaten the self‐concept by undermining perceived autonomy and evoking concerns about self‐presentation (Brooks et al. 2015; Tost et al. 2012). Furthermore, research indicates that decision‐makers tend to question the professional competence of advisors known for spreading negative gossip, consequently reducing their likelihood of seeking advice from them (Gordon and Schweitzer 2024).

Since advice taking is a process of interpersonal interaction between advisors and decision makers, it is inevitably related to impression formation and impression management. Impression formation serves as both the premise and the goal of impression management. People form impressions of others based on not only their behaviours (Carlston and Skowronski 1994), but also overt decision‐making or advice taking, even when it comes to impressions of Artificial Intelligence (McKee et al. 2024).

The Stereotype Content Model (SCM) proposes that the formation of impressions about others is typically based on two fundamental dimensions: warmth and competence (Brambilla et al. 2011; Fiske et al. 2002). Warmth is viewed as a communal dimension, reflecting the desire to connect and cooperate with others (Cuddy et al. 2008). Warm traits mainly include specific attributes such as sincerity, friendliness, and trustworthiness. Competence is viewed as an agentic dimension, reflecting the ability to achieve and respond effectively to challenges. Competence mainly includes exhibiting efficiency, skill, and intelligence (Fiske et al. 2002; Kervyn et al. 2009; Rom et al. 2016; Rom and Conway 2018).

Regarding the relationship between advice interaction and making a competent versus warm impression, few studies have examined this topic, and the conclusions are inconsistent. Brooks et al.'s (2015) Stuties 1‐5 assessed how being asked for advice influences the advisor's perceptions of the advice seeker's competence. Their results show that the advisors perceive those who seek advice as more competent than those who do not; however, the decision makers have the opposite view (Pilot Studies A and B). Rosette et al. (2015) also found that male leaders who seek help will be evaluated as less competent than male leaders who do not seek help. Researches on advice taking suggest that powerless individuals are more likely to rely on advice, and advice use sends opposing signals to an advisor regarding the advisee's competence (Lee 2002; Palmeira and Lopez 2023; See et al. 2011; Tost et al. 2012). Schultze et al. (2018) examined the effects of decision‐maker agency and communion on advice taking in quantity estimation tasks. They found that agency reduced advice taking, mediated by individuals' perceptions of their own competence, whereas communion showed no consistent relationship with advice taking. Contrary to these results, Blunden et al.'s (2019) Study 2 found that those who sought advice but did not adopt it were rated as less warm and less competent by their advisor. Wice and Davidai (2020) presented participants with a vignette of someone who either conforms or refuses to conform and asked them to judge this person's character; participants viewed the nonconforming protagonists as more competent than the conforming protagonists and the nonconforming protagonists as less warm and tolerant than the conforming protagonists.

Clearly, the relationship between advice interaction and competence is different in the eyes of advisors and decision makers or third parties. In the advisor's view, seeking and taking advice improves their perception of the advice seeker's ability, but in the eyes of decision makers or third parties, seeking advice reduces their perception of the advice seeker's ability. Blunden et al. (2019) argued that when decision makers disregard the advice they receive, advisors' egos are likely to be threatened, and they reduce their feelings of threat by generating negative evaluations of the ego offender. Therefore, the advisor will judge seekers whom they perceive as ignoring or disregarding their advice as less warm and less competent than seekers who follow their advice. For decision makers, when they want to prove their ability by showing that their judgment is better than that of others, adopting advice poses a threat to their ego.

Furthermore, there is no direct research on the relationship between advice taking and warm impression currently. However, research on the influence factors of advice taking has found that decision makers who were happy (Yan et al. 2014) or grateful (de Hooge et al. 2014), in low power (See et al. 2011), similar to advisors (Gino et al. 2009) and trusting others (Milyavsky and Gvili 2024; Gino and Schweitzer 2008), exhibit a higher degree of taking advice, which means that taking advice makes the decision maker appear more genuine, agreeable, cooperative, trusting of others, and with a high desire of social connection with others. These traits are considered reliable indicators of interpersonal warmth. Based on this reasoning, we propose the following hypotheses:

Hypothesis 1 *Advice‐taking behaviour influences impression formation. Specifically, decision makers who accept (*vs. *reject) advice will be perceived as* (*a*) *warmer but* (*b*) *less competent*.

Individuals engage in impression management precisely because they are aware that others form impressions of them (Bjornsdottir et al. 2022; Engstrom et al. 2024). This ability to infer and understand how others form one's impressions based on the outcomes of one's decisions is known as meta‐perception or as judgements on “how others view us” (Ohtsubo et al. 2009; Toma and Carlson 2015; Rom et al. 2016; Rom and Conway 2018). Based on previous studies, we expect that decision makers can accurately perceive others' impressions of them through their advice taking, where decision makers who accept other's advice are perceived as warmer and less competent than those who reject others' advice.

Hypothesis 2 *Decision makers possess accurate meta‐perceptions. That is, their beliefs about how others judge their* (*a*) *warmth and* (*b*) *competence based on their advice‐taking will align with the actual impressions formed by others*.

### 1.2 Impression Management and Advice Taking

Impression management refers to the process by which individuals consciously monitor and regulate their speech, behaviour, appearance, or expressions to influence others' perceptions of them, thereby shaping a desired social image (Goffman 1959; Leary 1995). Leary (1995) proposed that impression management comprises two distinct processes: impression motivation and impression construction. Impression motivation primarily aims to gain social approval, acquire power and influence, and maintain self‐concept. Impression construction refers to the specific strategies and manifestations employed in impression management. Jones and Pittman (1982) identified five categories of impression management strategies: ingratiation, self‐promotion, exemplification, supplication, and intimidation. Most research on impression management behaviours at work has focused on self‐promotion tactics, such as self‐enhancement or ingratiation (Bolino et al. 2008). People are more motivated to control how others perceive them when they believe that their public images are relevant to the attainment of desired goals.

Scarcely any research, however, has examined tactics aimed at advice taking. Since individuals are aware that others form impressions of them based on the results of their decisions, they strategically adjust their decision‐making strategies to make an ideal impression and in turn achieve their goals (Rom et al. 2016; Rom and Conway 2018). For example, in the trolley dilemma, Rom and Conway (2018) found that decision makers are more likely to accept killing one person instead of five people when they expect to be evaluated as competent and less likely to accept doing so when they expect to be evaluated as warm. We expect that the meta‐perception of how others infer their warmth and competence from the degrees of advice taking will prompt decision makers to strategically adopt advice from others to achieve their goals.

Hypothesis 3 *Impression management goals influence advice‐taking. When motivated to appear warm (*vs. *competent), decision makers will increase their degree of advice taking*.

However, impression management is context‐dependent. According to impression management theory, the motivation to manage one's impression is heightened when the target of the impression holds significant influence over the achievement of an individual's goals (Goffman 1959; Leary 1995). Therefore, we propose that the source of advice serves as a critical moderator: when advice comes from a high‐value target, individuals are more motivated to carefully tailor their self‐presentation for this influential “audience,” thereby amplifying the strategic behaviour outlined in Hypothesis 3.

Hypothesis 4 *The source of advice moderates the effect proposed in* H3. *The relationship between impression motivation (warmth* vs. *competence) and advice‐taking will be stronger when the advice comes from a high‐value target compared to a low‐value target*.

### 1.3 The Present Study

A critical issue addressed by the present work concerns the impression management mechanisms underlying advice‐taking. That is, people may strategically adapt their degree of advice taking to present themselves favorably. We conducted our research mainly with a simulated job interview situation (except Study 1b), which is “low‐hanging fruit” for impression management scholars (Liljenquist 2010). Most impression management studies have capitalized on the opportunity presented by this situation to examine reactions to various self‐presentation strategies (Bolino et al. 2008).

To demonstrate whether the decision maker will strategically use advice‐taking as a means of impression management, we need first to find out whether decision makers recognize that other people form an impression of them based on their advice taking. Accordingly, Study 1 was aimed to examine whether individuals explicitly form an impression according to degrees of advice taking, with those who follow advice being considered warmer and those who reject it being considered more competent, and the accuracy of participants' meta‐perceptions.

**FIGURE 1:**
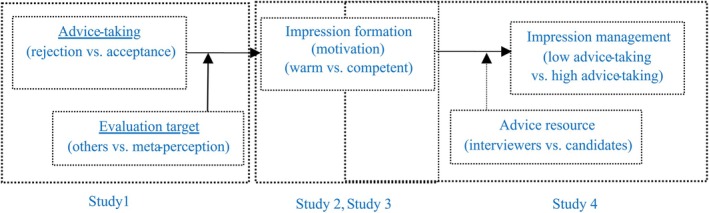
The conceptual model.

In Study 2 and positions in Study 3, social role expectations were manipulated through different instructions to investigate whether individuals adjust their receptivity to advice depending on whether warmth or competence is preferred. If there is an impression management mechanism in advice taking, then the factors that affect impression management will also affect advice taking. To further examine the impression management mechanism in advice taking, Study 4 was designed to explore whether participants adopted advice strategically when they were informed that the advice came from interviewers or from other candidates. The conceptual path diagrams for the four studies are presented in Figure 1.

In the present study, we used the Judge–Advisor System (JAS) paradigm, which has been used in many advice taking studies (for a review, see Bonaccio and Dalal 2006). And in Studies 2, 3, and 4, we used the weight of advice (WOA, Harvey and Fisher 1997; Yaniv and Foster 1997) to assess the degree of advice taking: $\mathrm{WOA}=\frac{\left(\text{finalestimate}-\text{initialestimate}\right)}{\left(\text{advice}-\text{initialestimate}\right)}$. A higher WOA value reflects a greater degree of advice taking. The WOA measure ranges from zero, which indicates that the advice had no impact on the individual's final estimate, to one, which indicates that the final estimate is consistent with the advice. When the initial estimation was equal to the adviser's estimation, the WOA value was undefined. We treated these observations as missing values. In the present study, if the subjects had a WOA > 1 in more than three rounds of estimation tasks, they were removed. We used the average of the WOAs to create an index of the extent to which the participant took advice.

We estimated the sample sizes of the four studies using Gpower3.1. With a medium effect size *f* = 0.25, we based our calculation on a 2 × 2 between‐subjects ANOVA design in Study 1a; the sample size estimated by G*power is 128 participants (32 for each group) to reach a power of 0.80. Using a 2 × 2 × 2 between‐subjects ANOVA design, in Study 1b, we calculated our target sample size; we required approximately 200 participants (25 for each group) to reach a power of 0.80. For Studies 2 and 3, we based our calculation on a one‐way ANOVA design, finding that reaching sufficient power (0.80) required at least 128 participants (64 for each group). For Study 4, we based our calculation on a 2 × 2 between‐subjects ANOVA design; the sample size estimated by G*power was 128 participants (32 for each group) to reach a power of 0.80. All experiments were approved by the Human Research Ethics Committee of University of Jinan (UJN2023‐032), and written informed consents were obtained from all subjects. All participants received a gift worth 3 Chinese yuan after the experiment.

## 2 Study 1

### 2.1 Study 1a

Study 1a examined whether participants were able to perceive others' impressions of them according to participants' degrees of advice taking (i.e., meta‐perception).

#### 2.1.1 Method

##### 2.1.1.1 Participants and Design

A total of 128 undergraduate students were recruited. The participants were randomly assigned to one of four conditions of a 2 (advice taking result: rejection vs. acceptance) × 2 (evaluation target: others vs. meta‐perception) between‐subjects design. Three participants were excluded because two of them chose 7 to evaluate the decision maker on all 9 traits of warmth and competence, while one of them chose 1 to evaluate the decision maker on all 9 traits of warmth and competence, leaving a final sample of 125 individuals (86 females, *M*_age_ = 21.05, SD = 1.88).

##### 2.1.1.2 Procedure and Materials

All participants first read a script about someone named Zhang (or themselves) applying for a job as a fitness coach. During the interview, the applicant was required to estimate the calories of three types of food. After making the initial estimate, they could modify their estimate according to the advisor's advice. The script included the applicant's initial decision, the advisor's advice, and the applicant's final decisions, indicating they refused or accepted advice. In the script, we predefined the calorie data of the applicant's initial estimates, advisor's advice (initial estimate × (1% ± 50%)), and the applicant's final estimates. Calorie data were downloaded from a professional weight management website (www.boohee.com).

A script of the advice rejection‐others (meta‐perception) condition is as follows:

Zhang applied for a staff member position at a Fitness Coach Training Institute. One of the interview questions involved estimating the calories of food with another candidate. One of the candidates is the decision maker, who is responsible for giving the final answer, and the other is the advisor, who is responsible for providing suggestions. The task includes three rounds. This time Zhang is the decision maker.

The first round: Please estimate how many calories are in 100 g of corn:

Zhang's (your) initial estimate: 100 Cal.

Advice from his (your) advisor: 150 Cal.

Zhang's (your) final estimate: 100 Cal.

In the advice rejection condition, the final decision was completely consistent with decision makers' initial estimation, whereas in the advice acceptance condition, it was completely consistent with the advisor's advice, which was 150 Cal in this example.

After reading this script, the participants in the *other* condition were asked to evaluate the warmth and competence of the decision maker from the perspective of bystanders. The participants in the *meta‐perception* condition were asked to imagine how a bystander would evaluate them in terms of warmth and competence when he read about the entire advice taking process. Measures of warmth and competence were adopted from Fiske and her colleagues (2002) and included nine items: four warmth traits (warm, good‐natured, tolerant, and sincere) and five competence traits (competent, confident, independent, competitive, and intelligent). The participants were asked to describe the decision maker on 7‐point scales ranging from 1 (*not at all*) to 7 (*very much*). The Cronbach's alpha coefficient of the four warmth traits was 0.76 (others)/0.93 (meta‐perception), and that of the five competence traits was 0.85 (others)/0.95 (meta‐perception). The data used to support all findings of this study are available from: https://figshare.com/account/articles/27916602?file=50833533.

#### 2.1.2 Results

The means and standard deviations of the warmth and competence ratings are shown in Table 1.

**TABLE 1:** The means and standard deviations of warmth and competence ratings (*M ±* SD).

		Warmth	Competence
Advice rejection	Other	3.38 ± 1.18 (*n* = 35)	5.31 ± 1.22 (*n* = 35)
	Meta‐perception	3.21 ± 1.46 (*n* = 32)	5.68 ± 1.94 (*n* = 32)
Advice acceptance	Other	5.21 ± 1.28 (*n* = 28)	2.59 ± 1.56 (*n* = 28)
	Meta‐perception	5.43 ± 1.43 (*n* = 30)	2.80 ± 1.36 (*n* = 30)

We applied warmth and competence ratings to two 2 × 2 ANOVA separately.

(1) Warmth ratings as the dependent variable.

The main effect of advice taking was significant *F* (1, 121) = 70.62, *p* < 0.001, η_*p*_^2^ = 0.37. The scores for the *advice acceptance* condition were significantly higher than those of the *advice rejection* condition. We found no main effect of evaluation target, *F* (1, 121) = 0.01, *p* = 0.91, η_*p*_^2^ = 0.00. The rating showed no significant differences across the other and meta‐perception conditions. Interactions were also found to be insignificant, *F* (1, 121) = 0.65, *p* = 0.42, η_*p*_^2^ = 0.005.

(2) Competence ratings as the dependent variable.

A main effect of advice taking was found, *F* (1, 121) = 174.69, *p <* 0.001, η_*p*_^2^ = 0.59. The scores of the *advice rejection* condition were significantly higher than those of the *advice acceptance* condition. We found no main effect of evaluation targets, *F* (1, 121) = 1.88, *p* = 0.17, η_*p*_^2^ = 0.15. The competence ratings showed no significant differences across the other and meta‐perception conditions. Interactions were not found to be significant, *F* (1, 121) = 0.15, *p* = 0.70, η_*p*_^2^ = 0.001.

#### 2.1.3 Discussion

Study 1a shows that advice taking affects individuals' evaluations and meta‐perceptions of decision makers and that there is no essential difference between the two forms of evaluation. Participants viewed decision makers who accepted others' advice as warmer and those who rejected others' advice as more competent. In the meta‐perception condition, participants thought others would rate them similarly to how they rated others. This means that decision makers exhibit a basic ability to predict or perceive other individuals' impressions of themselves through advice taking.

### 2.2 Study 1b

In Study 1a, participants in the meta‐perception condition also received the information as if they themselves had made the final estimate of 100 Cal. In fact, however, they had no chance to make the decision themselves. Although this design increases experimental control, participants may not really see this decision as “their own decision” in the meta‐perception condition. Therefore, in Study 1b, instead of an estimation task, we use choice tasks to describe the situation in which the decision maker straight‐lined their responses: accept or reject. In Study 1a, the participants rated both warm and competency traits, which may cause a demand effect, that is, participants may assume if someone is highly competent, the researcher expects them to say the person is less warm. Thus, a between‐subjects design was used to rule out this possibility where half the participants rate warmth, and half rate competence.

#### 2.2.1 Method

##### 2.2.1.1 Participants and Design

A total of 392 undergraduate (76 males, *M*_age_ = 19.47, SD = 0.88) took part in this study. All the participants were from three classes of a psychology elective course. All participants received course credit for their participation. Participants were randomly assigned to one of eight conditions in a 2 (advice taking result: rejection vs. acceptance) × 2 (evaluation target: other vs. meta‐perception) × 2 (trait: warm vs. competency) between‐subjects design.

##### 2.2.1.2 Procedure and Materials

The participants were informed the following: “This is a survey of the relationship between behaviour and personality. Everyone has their own behaviour styles. We can infer someone's personality from their behaviour. In psychology class, the teacher presented two behaviours by Li Yang and asked everyone to speculate about his personality.”

Then, participants read the description of Li Yang, where the decision maker either adopted or refused the other's advice.

Li Yang participated in a TV quiz show. According to the program rules, the competitor is allowed to seek off‐site help once during the answer process. However, to prevent the advisor searching for the answer from the Internet in advance, the program stipulates that the competitor can only call a relative or friend who does not watch the program for help (therefore, relatives and friends do not know the contestant's final choice). Li Yang requested off‐site help when encountering the following questions:

What is the name of the first nuclear‐powered Mars rover in the United States that successfully landed on Mars in August 2012?

A. Pathfinder B. Curiosity.

Li Yang was not sure whether to choose A or B, so he called his friends abroad. His friends suggested choosing B. Li Yang finally chose B (A).

After the show, Li Yang won a 3‐day, 2‐night tourism package. There are two tourist destinations to choose from:

A. Sanya, Hainan B. Xishuangbanna, Yunnan.

Li Yang was not sure which destination to choose. The TV staff suggested Xishuangbanna. Li Yang decided to think about it. However, finally, he adopted the suggestions of the staff and chose Xishuangbanna (Sanya, Hainan).

After reading this passage, participants were asked to evaluate the warmth and competence of the decision maker. Measures of warmth and competence were the same as in Study 1a. The Cronbach's alpha coefficient of the four warmth traits was 0.91, and that of the five competence traits was 0.88.

Participants in the others condition were presented with the following:

Based on this information, what do you think about Li Yang? From your perspective, how do you evaluate Li Yang's behaviour in terms of the following traits?

Participants in the meta‐perception condition was presented with the following:

As often happens, there was misinformation, and the teacher mistakenly wrote your name instead of Li Yang's name. Thus, the other person thinks you really participated in the program. Now, take a moment to imagine that another person saw the choices you made. Based on that information, what would they think about you? From their perspective, how well do you think they would say each trait describes you?

#### 2.2.2 Results

The means and standard deviations of the warmth and competence ratings are shown in Table 2.

**TABLE 2:** The means and standard deviations of warmth and competence ratings (*M ±* SD).

		Warmth	Competence
Advice rejection	Other	3.67 ± 1.24 (*n* = 52)	4.40 ± 0.91 (*n* = 51)
	Meta‐perception	3.88 ± 1.53 (*n* = 50)	5.08 ± 1.16 (*n* = 49)
Advice acceptance	Other	4.89 ± 1.05 (*n* = 45)	3.60 ± 1.00 (*n* = 48)
	Meta‐perception	5.00 ± 1.17 (*n* = 47)	4.02 ± 1.55 (*n* = 50)

We applied a 2 × 2 × 2 ANOVA. The main effect of the evaluation target is significant, *F* (1, 384) = 8.17, *p* = 0.004, η_*p*_^2^ = 0.02. The main effect of advice taking and trait is not significant, *F* (1, 384) = 0.91, *p* = 0.34; *F* (1, 384) = 0.46, *p* = 0.50. The interaction of advice taking and trait is significant, *F* (1, 384) = 72.56, *p* < 0.001, η_*p*_^2^ = 0.16. Other interactions are not significant. Then, we reported the results with warmth and ability as dependent variables.

(1) Warmth rating as the dependent variable.

The main effect of advice taking was significant *F* (1, 190) = 41.40, *p* < 0.001, η_*p*_^2^ = 0.18. The scores of the *advice acceptance* condition were significantly higher than those of the *advice rejection* condition. We found no main effect of evaluation target, *F* (1, 190) = 0.76, *p* = 0.38, η_*p*_^2^ = 0.004 (negligible effect), 95% CI [0.000, 0.026], observed power = 0.14. There were no significant differences in rating across Other and Meta‐perception conditions. Interactions were also found to be insignificant, *F* (1, 121) = 0.07, *p* = 0.79, η_*p*_^2^ < 0.001, 95% CI [0.000, 0.041], observed power = 0.06.

(2) Competence rating as the dependent variable.

A main effect of advice taking was found, *F* (1, 194) = 31.11, *p <* 0.000, η_*p*_^2^ = 0.14. The scores of the *advice rejection* condition were significantly higher than those of the *advice acceptance* condition. We also found a main effect of evaluation targets, *F* (1, 194) = 10.68, *p* = 0.001, η_*p*_^2^ = 0.05. The scores of meta‐perceptions were significantly higher than those of other conditions in the competence ratings. Interactions were not found to be significant, *F* (1, 194) = 0.59, *p* = 0.44, η_*p*_^2^ = 0.003, 95% CI [0, 0.023], observed power = 0.09.

#### 2.2.3 Discussion

The interaction between advice‐taking and evaluation target (other‐perception vs. meta‐perception) in Studies 1a and 1b was not statistically significant. This pattern suggests that people's judgments of others who take advice and their beliefs about how others would judge them tend to align, reflecting a shared normative understanding of the social meaning of advice‐taking. Such perceptual alignment offers a plausible basis for strategic impression management: when individuals assume that others evaluate advice‐taking similarly to how they themselves do, they are better positioned to regulate their behaviour to influence others' impressions. At the same time, we acknowledge that the non‐significant interaction may also reflect limited statistical power or a small underlying effect; therefore, we report effect sizes and confidence intervals to allow readers to assess the precision of these estimates. Next, we will explore whether people adjust their degree of advice‐taking strategically to manipulate others' impression of them because of this awareness.

## 3 Study 2

In Study 2, we examined whether people sometimes strategically adjust their degree of advice taking to conform with social expectations. In a hypothetical job interview scenario, we randomly assigned participants to warm requirements and ability requirements conditions. The participants were asked to estimate the calories in food according to others' advice. If they realize that the job requires the applicant to be warm, they will adopt the other people's advice more than if they realize the job requires the applicant to be competent.

### 3.1 Method

#### 3.1.1 Participants and Design

We recruited 134 undergraduate and postgraduate students (76 females, *M*_age_ = 21.22; SD = 2.57). No data were excluded. The participants were randomly assigned to one of two conditions of a single‐factor (expected impression: warm vs. competent) between‐subjects design. The dependent variable was WOA.

#### 3.1.2 Procedure and Materials

We seated participants in separate rooms in front of computers. We presented all of the instructions and tasks on the computer screen. As a cover story, we told participants that the purpose of this study was to investigate the understanding of various groups of people about the workplace.

The participants in the warmth (competence) condition were presented with the following instructions:

Imagine that you are interviewing for a position at the Fitness Coach Training Institute and that you really hope to get the job. The ideal staff member should be warm, friendly, tolerant and sincere (competent, confident, independent, competitive, and intelligent). One of the interviews involves estimating the number of calories in certain foods whereby other candidates can provide advice. The estimation task involves ten rounds.

The calorie estimation task (Yaniv and Choshen‐Hillel 2012) followed the JAS paradigm. In each round of the calorie estimation task, the participants were first presented with the name of a certain food item and asked to make an initial estimate of the number of calories it contained (e.g., “Please estimate the number of calories in 100 g bananas”). After they made their initial estimate, another candidate's opinions were presented. The advice was simplified and referred to as “opinion from a candidate” without additional information. They were asked to make a final decision based on this guidance. Importantly, the advice values were calculated in real time according to the initial estimates recorded by each computer and were determined using the following formula: advice = participant's initial estimate × (1% ± 50%). When a participant underestimated relative to the true value, 50% was added to the initial estimate; when a participant overestimated relative to the true value, 50% was subtracted. Consequently, advice generally pointed in the direction of the true value (Hütter and Ache 2016). A value (initial estimate × 2%) was also randomly added or removed as noise to conceal the artificial nature of the advice.

### 3.2 Results

A one‐way analysis was conducted using WOA as the dependent variable. The results showed the main effect of expected impressions was significant, where *F* (1, 132) = 19.15, *p* < 0.001, η_*p*_^2^ = 0.13. The mean WOA of the warmth condition (*M* = 0.55, SD = 0.24) was significantly higher than that of the competence condition (*M* = 0.37, SD = 0.25).

### 3.3 Discussion

The results of this study suggest that the decision makers strategically adopt others' advice to engage in impression management. When a warm impression must be made, a decision maker will adopt more advice than when an impression of competence must be made.

## 4 Study 3

In organizations, the requirements for competence vary with position. Some positions focus on competence (e.g., a combat commander), while others focus on warmth, and individuals have a certain understanding of the characteristics of various positions. This study explored whether participants strategically took others' advice according to their understanding of the positions for which they were interviewing. We expected that when informed that they were applying for a position focused on warmth, our participants would follow more advice than when told that they were applying for a position focused on competence.

### 4.1 Method

#### 4.1.1 Participants and Design

A total of 136 undergraduate students participated in this Study. The participants were randomly assigned to one of two conditions of a single‐factor (expected position: head of a town vs. neighbourhood committee) between‐subjects design. Seven participants were excluded because their WOA was > 1 in more than 3 trials. Finally, there were 129 valid participants (82 females, *M*_*age*_ = 21.38, SD = 1.74).

#### 4.1.2 Procedure and Materials

Before testing our main hypothesis, we conducted a pretest to confirm that the positions used in the study indeed captured the stereotypes of warmth and competence. We recruited 60 participants (30 females) to complete a five‐minute survey. As three participants did not complete the scale, a total of 57 valid data sets were obtained. We presented the participants with our four pairs of matching positions (see Table 3): 1. the head of a town committee and the head of a neighbourhood committee; 2. a financial consultant and a nutrition consultant; 3. a company owner and a company instructor; and 4. an investment counsellor and a psychological consultant. In each pair, the first positions were competence‐oriented, while the latter were warmth‐oriented positions. The participants were asked to evaluate the 8 positions in terms of four warmth traits (warm, good‐natured, tolerant, and sincere) and five competence traits (competent, confident, independent, competitive, and intelligent) on 5‐point scales of 1 (*not at all*) to 5 (*very much*) (Fiske et al. 2002). We aimed for the positions of warmth and competence to be matched, and each position had a large difference in warmth and competence scores. Therefore, for competence‐oriented positions, upon subtracting the warmth score from the competence score, larger values indicated the position to be more competence‐oriented. For warmth‐oriented positions, upon subtracting the competence score from the warmth score, larger values indicated the position to be more warmth‐oriented. In the four pairs of matching positions, the head of a town was rated as more competent than warm, and the head of a neighbourhood committee was rated as more warm than competent. So the town and neighbourhood committee leader positions were ultimately used for the formal experiment.

**TABLE 3:** Ratings of 4 pairs of positions on warmth and competence traits.

Competence positions	Warmth	Competence	Competence‐warmth	Warmth positions	Warmth	Competence	Warmth‐competence
Head of a town committee	829	1176	347	Head of a neighbourhood committee	1017	940	77
Financial consultant	719	1222	503	Nutrition consultant	896	1040	−144
Company owner	705	1229	524	Company instructor	820	1139	−319
Investment counsellor	718	1244	526	Psychological consultant	1068	1168	−100

In the formal experiment, the participants were told that they were to participate in a simulated interview task. The position interviewed for was the head of a town (or neighbourhood committee) and that a calorie estimation task was included in the interview. They were asked to complete the estimation task in reference to advice from others (same as Study 2). There were five estimated tasks in this study.

### 4.2 Results

A one‐way analysis was conducted using position as the independent variable and WOA as the dependent variable. The results showed the main effect of position was found to be significant, *F* (1, 127) = 6.79, *p* = 0.010, η_*p*_^2^ = 0.05. The mean WOA for applying for the neighbourhood committee leader role (*M* = 0.42, SD = 0.22) was significantly higher than that for the town committee leader role (*M* = 0.31, SD = 0.23).

### 4.3 Discussion

This study found that the participants strategically took advice during the interviews based on their understanding of the position they were applying for. This result replicated and expanded the findings of Study 2. By having two experimental groups apply for positions that only implicitly require different levels of warmth and competence, this study reduced the demand characteristics compared to Study 2.

In Study 4, we examined a potential moderator, the advice sources. Specifically, do participants take advice more when they know the advisor is from the interviewer?

## 5 Study 4

In this Study, we explored whether the degree of advice taking was affected by the source of the advice (interviewers or other candidates). In an interview situation, an interviewer's impression of the interviewee determines whether they are employed or not. The interviewers are the people interviewees  most want to impress. Therefore, we speculated that in the process of impression management, advice from interviewers may have a greater effect than advice from other candidates.

### 5.1 Method

#### 5.1.1 Participants and Design

A total of 220 undergraduate and postgraduate students participated in the study. They were randomly assigned to one of the conditions of a 2 (expected impression: warm vs. competent) × 2 (advice sources: interviewer, other candidate) between‐subjects design. Eight participants were removed for having WOAs > 1 over three rounds, resulting in a final sample of 212 (126 females, *M*_age_ = 20.50, SD = 1.80).

#### 5.1.2 Procedure and Materials

In this study, the participants were also told that each round of the calorie estimation task included an initial estimate and a final estimate. After they made their initial estimate, others' advice was presented. They were asked to make a final decision based on this guidance. Different from studies 2 and 3, the participants were told that there were two advice pools: “one is from other candidates who have just completed the task, and the other is from the interviewer who also estimated the task before the interview in order to understand the task. Here is a random program to help you select an advice pool. Please press the ENTER key to start.” It is worth noting that the random program was designed only to present the interviewers' advice pool to the participants in the interviewer advice condition, and the opposite is true for the other‐candidate advice group. In fact, the advice from the two pools was the same and was manipulated just as in study 2.

### 5.2 Results

The mean and standard deviation of the WOA are shown in Table 4.

**TABLE 4:** The means and standard deviations of the WOA (*M* ± SD).

	Interviewer	Other candidate
Warm	0.68 ± 0.21 (*n* = 52)	0.55 ± 0.25 (*n* = 52)
Competent	0.47 ± 0.20 (*n* = 54)	0.47 ± 0.21 (*n* = 54)

A two‐way ANOVA was conducted using WOA as the dependent variable. The results showed that the main effect of expected impressions was significant, where *F* (1, 208) = 22.96, *p* < 0.001, η_*p*_^2^ = 0.10. The main effect of advice source was significant, where *F* (1, 208) = 4.60, *p* = 0.033, η_*p*_^2^ = 0.02. The interaction of expected impressions and advice sources was significant, where *F* (1, 208) = 4.42, *p* = 0.037, η_*p*_^2^ = 0.02. The simple effect analysis showed that, for the interviewer's advice, the participants seeking to make a warm impression were significantly more likely to adopt the advice than those seeking to appear competent, *F* (1, 208) = 23.76, *p* < 0.001, η_*p*_^2^ = 0.10; for the other interviewee's advice, the advice taking rate also differed between those seeking to make a warm impression and those seeking to appear competent, *F* (1, 208) = 3.62, *p* = 0.059, η_*p*_^2^ = 0.02.

### 5.3 Discussion

The results of this study are basically consistent with those of Studies 1–3. Participants were more likely to adopt advice strategically. The results of this study also show that the advice source plays a moderating role in interviewees' impression management. When advice came from the interviewer, the degree of advice taking differed between those seeking to make a warm impression and those seeking to appear competent.

## 6 General Discussion

Across four studies, we proposed and tested an impression management account of advice‐taking. Drawing upon Goffman's (1959) impression management theory, we posited that advice‐taking is not merely a cognitive process aimed at accuracy, but a strategic social behaviour deployed to shape others' perceptions. Our findings demonstrate that decision makers perceive others' impressions of their warmth and competence based on the degree of advice taking (Study 1), and strategically adjust their advice taking to make the desired impression (Studies 2–4), particularly when the audience is motivationally relevant (Study 4). This program of research moves beyond the established literature on impression formation in advice‐taking to suggest an impression management mechanism affects the process of advice taking.

### 6.1 Main Findings

Our findings can be interpreted through a multi‐stage theoretical model of impression management theory.

First, the foundation of this process lies in shared social cognition, as captured by the Stereotype Content Model (SCM; Fiske et al. 2002). Study 1 provides robust evidence that the act of advice‐taking is universally interpreted through the fundamental dimensions of warmth and competence. Decision makers who accepted others' advice are perceived as warmer—conveying trust, cooperativeness, and a desire for social harmony. In contrast, those who rejected others' advice are perceived as more competent—signaling independence, confidence, and self‐reliance. This consensus in perception (across both “Other” and “Meta‐perception” conditions) is the mapping between behaviour (advice acceptance/rejection) and trait inference (warmth/competence). Therefore, they may strategically adjust their degrees of advice taking in the interest of impression management.

Second, Studies 2 and 3 move beyond recognition to demonstrate action. Here, the decision of whether to take advice is driven by impression motivation (Leary 1995)—the desire to be seen in a particular way to achieve a goal (e.g., getting a job). When the goal is to appear warm, decision makers strategically increase their advice‐taking. This is similar to the results of ingratiation strategies (Bolino et al. 2008)—a strategy aimed at being perceived as likable and agreeable. By deferring to others' opinions, individuals signal that they value connection and harmony, effectively managing their image to fit warmth‐oriented roles. Conversely, when the goal is to appear competent, decision makers strategically decrease their advice‐taking, which indicates that they favour their own point of view and are not influenced by others. Thus, decision makers may use a form of self‐promotion strategies to be viewed as more competent.

Finally, the strategic use of this tool is context‐dependent. Studies 4 was designed to explore when people do and do not engage in impression management in advice taking. Therefore, we examined one potential moderator, the advice sources, which has been demonstrated to affect impression management in the field of social cognition. When the participants were informed that the advice came from the interviewers (who hold power over the decision maker's outcomes) and not from other candidates, they were more likely to adopt advice. It can be considered that, when advice comes from interviewers, the participants' motivation for impression management is amplified. This further promotes the understanding of the impression management side of advice taking.

### 6.2 Theoretical Contributions

First, we propose an impression management mechanism of advice taking. The degree of advice adoption serves as a flexible tool for decision‐makers to engage in impression management. Increasing advice adoption resembles the use of “ingratiation” strategies, demonstrating approachability and willingness to cooperate to project an image of warmth; whereas decreasing advice adoption parallels “self‐promotion” strategies, highlighting personal competence by showcasing independence and confidence. This expands our understanding of impression management behaviours, extending it from traditional verbal self‐presentation to social interactions within the decision‐making process.

Second, researchers believe that individuals have accurate meta‐perceptions of others' impressions of themselves and that decision makers predict how others will evaluate them based on the results of their decisions (Ohtsubo et al. 2009; Rom et al. 2016; Rom and Conway 2018). The present study extends this finding to the domain of advice taking. Decision makers can also accurately predict others' impressions of their warmth and competence based on their advice taking tendencies. This predictive capacity is not rare but rather something that most individuals possess.

Third, previous research on advice taking has focused mainly on the advice discounting effect and its mechanism, such as anchoring (Tversky and Kahnerman 1974), differential information (Yaniv and Kleinberger 2000), and egocentric bias (Krueger 2003). Our research demonstrates that regardless of whether the degree of advice taking is high or low, it can serve the function of impression management. High adoption is likely a sign of a desire to appear warm, while low adoption is likely a sign of a desire to appear competent. Our findings highlight an interesting point in relation to interpreting the high and low degrees of adoption (Gino 2008).

### 6.3 Practical Implications

Our findings offer several implications for organizational practices and interpersonal dynamics.

First, for organizations and managers, the findings of this study hold significant value for enhancing the effectiveness of recruitment, training, and internal communication. Recruitment leads and business managers should recognize that an interviewee's level of advice‐taking not only reflects their cognitive style or openness to information but may also constitute a strategic form of self‐presentation. This awareness can help mitigate potential biases in evaluation.

Second, for interpersonal interactions in broader contexts, understanding this dynamic mechanism can improve communication quality in any relationship involving impression management (e.g., such as teacher‐student or doctor‐patient relationships). Individuals in social interactions can also more clearly examine their own goals, autonomously choosing whether to use advice‐taking as a bridge to build relationships (by accepting advice) or as a means to establish professional credibility (by critically evaluating advice).

### 6.4 Limitations and Future Research Directions

From a social cognition perspective, the current work proposes and examines a mechanism of impression management in advice taking. However, this study also has limitations.

First, regarding the advice‐taking task, this study employed a calorie estimation task. While the calorie estimation task seems appropriate for a job interview as a fitness coach (e.g., in Study 2), it is not very suitable for the interview as a head of a town and neighbourhood committee in Study 3. Other tasks, such as distance estimation and weight estimation, could be used in future research. Additionally, in the estimation tasks used in this study, none had objective answers and no incentives were implemented to motivate participants to make accurate estimates. This design may have amplified the influence of impression management motivation, since participants were not held accountable for decision accuracy; they could more easily focus on shaping their image through advice‐taking. If the tasks involved objective answers (e.g., estimating the distance between cities) and incorporated accuracy incentives (e.g., monetary rewards), decision‐makers might face a trade‐off between impression management and task performance. Under such conditions, the impact of impression management on advice‐taking could be attenuated, though it likely would not disappear entirely in interpersonal‐oriented contexts. Future research could adopt tasks with objective answers and incentives to further validate the robustness of the impression management mechanism.

Second, we embedded advice taking in the background of the interview. Applicants' impression management is the most widely existing phenomenon in actual job interviews. The applicants strategically adopt advice to elicit positive interviewer evaluations. However, impression management may occur within a broader context. Future research may thus explore whether the impression management mechanism of advice taking can be found in other interpersonal relationships, such as intimate, parent–child, mentoring, and doctor‐patient relationships.

Third, according to the “big two” dimension of social cognition, this study divided the decision maker's impression into competence and warmth, and lacked a neutral condition of reference, in which participants do not receive the instruction to optimise one of the two ratings. So we do not know what judges would have chosen outside of an impression management context. In addition, there can be many kinds of impressions, such as those based on morality. Even warmth and competence can be high or low. Future research should explore the influence of other expected impressions on advice taking.

Finally, we used WOA as advice taking index in Studies 2, 3, and 4, which has inevitable limitations. In future research, a new measure of advice utilization, such as IOA (Soll et al. 2022) or mixed‐effects regression coefficients (Rebholz et al. 2023) can be adopted.

## Ethics Statement

All survey and experiments were approved by the Human Research Ethics Committee of University of Jinan (UJN2023‐032), and written informed consent was obtained from all subjects. All participants received a gift worth 3 Chinese yuan after all the measures.

## Conflicts of Interest

The authors declare there are no conflicts of interest.

## Data Availability

The data that support the findings of this study are openly available in figshare at https://figshare.com/account/articles/27916602?file=50833533, reference number 27916602?file=50833533.
